# Normative Values for Sport-Specific Left Ventricular Dimensions and Exercise-Induced Cardiac Remodeling in Elite Spanish Male and Female Athletes

**DOI:** 10.1186/s40798-022-00510-2

**Published:** 2022-09-15

**Authors:** Araceli Boraita, Leonel Díaz-Gonzalez, Pedro L. Valenzuela, María-Eugenia Heras, Francisco Morales-Acuna, Adrián Castillo-García, María J. Lucia, Pedro Suja, Alejandro Santos-Lozano, Alejandro Lucia

**Affiliations:** 1 Department of Cardiology, Sports Medicine Center, Spanish Higher Sports Council, C/Pintor El Greco s/n, 28040 Madrid, Spain; 2 Cardiology Department, CEMTRO Clinic, Madrid, Spain; 3 Cardiology Department, La Paz Hospital, Madrid, Spain; 4 Research Institute of the Hospital, 12 de Octubre (“imas12”, PaHerg Group), Madrid, Spain; 5 Integrative and Neuromuscular Exercise Physiology Laboratory, Faculty of Medicine, School of Kinesiology, Universidad Finis Terrae, Santiago, Chile; 6 Fissac – Physiology, Health and Physical Activity, Madrid, Spain; 7 Departamento de Informática, Universidad Carlos III, Madrid, Spain; 8 i+HeALTH Research Group, Department of Health Sciences, European University Miguel de Cervantes, Valladolid, Spain; 9 Faculty of Sport Sciences, Universidad Europea de Madrid, Madrid, Spain

**Keywords:** Echocardiography, Sports cardiology, Pre-participation screening, Normative values, Athlete’s heart

## Abstract

**Background:**

There is debate about the magnitude of geometrical remodeling [*i.e.*, left ventricle (LV) cavity enlargement vs. wall thickening] in the heart of elite athletes, and no limits of normality have been yet established for different sports. We aimed to determine sex- and sport-specific normative values of LV dimensions in elite white adult athletes.

**Methods:**

This was a single-center, retrospective study of Spanish elite athletes. Athletes were grouped by sport and its relative dynamic/static component (Mitchell’s classification). LV dimensions were measured with two-dimensional-guided M-mode echocardiography imaging to compute normative values. We also developed an online and app-based calculator (https://sites.google.com/lapolart.es/athlete-lv/welcome?authuser=0) to provide clinicians with sports- and Mitchell’s category-specific Z-scores for different LV dimensions.

**Results:**

We studied 3282 athletes (46 different sports, 37.8% women, mean age 23 ± 6 years). The majority (85.4%) showed normal cardiac geometry, particularly women (90.9%). Eccentric hypertrophy was relatively prevalent (13.4%), and concentric remodeling or hypertrophy was a rare finding (each < 0.8% of total). The proportion of normal cardiac geometry and eccentric hypertrophy decreased and increased, respectively, with the dynamic (in both sexes) or static component (in male athletes) of the sport irrespective of the other (static or dynamic) component. The 95th percentile values of LV dimensions did not exceed the following limits in any of the Mitchell categories: septal wall thickness, 12 mm (males) and 10 mm (females); LV posterior wall, 11 mm and 10 mm; and LV end-diastolic diameter, 64 mm and 57 mm.

**Conclusions:**

The majority of elite athletes had normal LV geometry, and although some presented with LV eccentric hypertrophy, concentric remodeling or hypertrophy was very uncommon. The present study provides sport-specific normative values that can serve to identify those athletes for whom a detailed examination might be recommendable (*i.e.*, those exceeding the 95th percentile for their sex and sport).

**Supplementary Information:**

The online version contains supplementary material available at 10.1186/s40798-022-00510-2.

## Key Points

- The present study, which includes data from 3282 elite white athletes (mean age 23 ± 6 years, 37.8% women) of 46 different sports, suggests that the great majority (85.4%) of these athletes have normal LV geometry.
- Although some present with LV eccentric hypertrophy (13.4%), cardiac remodeling and concentric hypertrophy seem very uncommon (each < 0.8% of total).
- Sport-specific normative values of LV dimensions in elite white adult athletes are presented along with an online and app-based calculator, which can serve to easily identify those athletes for whom a detailed examination might be recommendable.

## Introduction

Participation in competition sports can induce cardiac tissue adaptations collectively known as “athlete’s heart.” Geometrical remodeling can affect the four cardiac chambers, with specific adaptations at the left ventricle (LV) level usually manifesting as increases in cavity size or wall thickness [1] of varying magnitudes that depend on several factors such as type of sport, sex, age, ethnicity, or years in competition [2, 3]. The resulting LV dimensions can exceed the limits expected for the general non-athletic population [4], which often makes it difficult to distinguish pathological from physiological alterations.

An increase in septal and posterior LV wall thickness (*i.e.*, ‘LV hypertrophy’) is commonly found in healthy athletes. Yet, according to a recent review from the American Society of Echocardiography (ASE), LV wall thickness rarely exceeds 13 mm or 11 mm in male and female athletes, respectively [2]. Some (2–18%) athletes, particularly black athletes, can have values > 13 mm [5, 6]. While controversial [4, 7, 8], an upper limit for LV wall thickness of 15 mm has been proposed for physiological sports-related LV hypertrophy [2, 7, 9]. There is also debate about the magnitude of LV cavity enlargement in athletes. Classical studies have reported values of LV end-diastolic diameter (LVEDD) > 55 mm in almost half of all athletes [10], but considerably higher values (60–70 mm) can be relatively prevalent in the athletic population (*i.e.*, 14%), particularly in those individuals with greater body surface area (BSA) [10].

Because cardiac structural adaptations are a function of the hemodynamic overload imposed to the heart during exertion, a main factor to be considered is the sport specialty in question, notably with divergent adaptations in endurance or strength-trained athletes engaged in purely ‘dynamic’ (*e.g*., distance running) or ‘static’ (*e.g.*, weight lifting, power lifting, bodybuilding) sports, respectively [11, 12]. It has been indeed classically assumed that an ‘endurance-trained’ heart would predominantly show eccentric LV hypertrophy (parallel increase in both LV mass and cavity, mainly due to volume overload and higher levels of diastolic wall stress), whereas a ‘strength-trained’ heart would show mostly concentric LV hypertrophy (increased LV wall thickness with essentially no increase in cavity size) [3]. To the best of our knowledge, however, there are no accepted upper normal limits for LV dimensions according to the different types of sport. In this regard, providing sport-specific normative values might be useful, notably to identify those athletes for whom a detailed examination might be recommendable (*e.g.*, those exceeding the 95th percentile for their sex and sport).

We aimed to describe LV dimensions in a large cohort of elite white adult athletes of both sexes categorized by their type of sport, and to propose normative values that can be used in clinical practice to identify athletes with non-physiological dimensions for their sex and sport specialty. Based on our experience, we hypothesized that only a minority of athletes would present LV dimensions characterized as ‘pathological' attending to established guidelines, and that cardiac remodeling characterized by excessive increases in LV wall thickness with no proportional changes in cavity dimensions would be less common among competitive athletes than previously thought, regardless of the sport.

## Methods

### Study Design and Participants

The present study followed a single-center, retrospective design and was conducted in the Cardiology Department of the Sports Medicine Center of the Spanish Higher Sports Council Spanish (Madrid, Spain). In this center, Spanish elite athletes participating in a broad range of sport disciplines and who are members of the national team in their specialty and compete in major international events (Olympics, and European and World championships) undergo routine, in-depth cardiological evaluation (one or more per year, most frequently during the preparatory mesocycle), including medical history, physical examination, anthropometric measurements, 12-lead electrocardiogram (ECG), exercise testing, and M-mode and Doppler two-dimensional (2D) echocardiography.

Data were retrospectively analyzed from athletes who had attended the center over a 17-year period (from the start of year 1997 to the end of 2013). Exclusion criteria included being nonwhite, having tested positive for the use of banned substances and/or suspended from participation in official competitions due to violation or anti-doping rules, structural cardiomyopathy, abnormal ECG findings (*i.e.*, not expected in athletes and suggestive of cardiomyopathy), sexual immaturity (< 18 and < 16 years for men and women, respectively), hypertension (baseline systolic or diastolic blood pressure ≥ 140 and ≥ 90 mm Hg, respectively), or an abnormal blood pressure response to exercise. For the sake of consistency, when this was possible, we attempted to choose for the present study in each athlete those evaluations corresponding to the aforementioned preparatory period (*e.g.,* usually during the fall for classical individual ‘Olympic’ sports such as track and field, swimming, or canoeing, among others or July–August for team ball sports). In those athletes with data available for more than one season, we used the evaluation from the last season because this was deemed to reflect the highest degree of adaptation to the sport in question. The study was approved by the local Ethics Committee (#1385226-1) and complies with the Declaration of Helsinki and its later amendments. Oral or written consent was obtained from all participants.

Athletes were categorized according to the modified Mitchell classification into nine groups attending to the relative dynamic/static component of their sport specialty, as recently done by us for normative values of aortic root dimensions (with the inclusion of some sports not included in the original Mitchell’s classification, *i.e.*, mountaineering, freestyle skiing, indoor soccer, motorboat racing, modern pentathlon, and water polo) [13]:

- IA, low static (< 20% of maximum voluntary contraction [MVC] and low dynamic (< 40% of maximum oxygen uptake [VO_2max_]) component
- IB, low static and moderate dynamic (40–70% VO_2max_)
- IC, low static and high dynamic (> 70% VO_2max_)
- IIA, moderate static (20–50% MVC) and low dynamic
- IIB, moderate static and moderate dynamic
- IIC, moderate static and high dynamic
- IIIA, high static (> 50% MVC) and low dynamic
- IIIB, high static and moderate dynamic
- IIIC, high static and high dynamic.

### Measures

Echocardiography evaluations were conducted using a Toshiba SSH-140A system (Toshiba Medical Systems, Tochigi, Japan) equipped with 2.5- and 3.75-MHz probes, or a Phillips Sonos 7500 system (Advance Diagnostics, Palo Alto, CA) equipped with a color, tissue Doppler, multifrequency 2–4 MHz transducer. All measurements were taken independently by two experienced sonographers (AB and MEH, 15 years working together). All LV dimensions were measured using 2D-guided M-mode imaging following ASE recommendations [14]. All participants were assessed under resting conditions (*i.e.*, during morning hours or early afternoon, after a rest period from the last exercise training session of at least 12 h). Height and weight were measured (accuracy of 0.1 cm and 0.1 kg, respectively) for the computation of BSA (see below).

Septal wall thickness (SWT), LV posterior wall thickness (LVPW), LVEDD, and LV end-systolic diameter (LVESD) (all in mm) were measured in the parasternal long-axis view, directly from the screen using the scale of the device itself, with the 2D-guided M-mode approach in real time and also guided by the ECG signal in bipolar lead CM5. All the echocardiographic measures corresponding to diastole and systole were obtained coinciding with the start of the QRS complex and with the maximal posterior displacement of the interventricular septum, respectively. Special care was taken when measuring SWT and LVPW to avoid including as part of the wall the different trabeculae both from the LV (false or ‘true’ tendinous chords) and right ventricle (mitral subvalvular apparatus and moderator band (or ‘septomarginal trabecula’)), because inclusion of these structures could erroneously reflect LV hypertrophy. All LV measures were obtained using the mean of three (or five, in case of doubt) cardiac cycles. We used the following equations to measure LV end-systolic volume (LVSV), LV end-diastolic volume (LVEDV), LV mass and LV ejection fraction (LVEF), respectively: LVESV (mL) = [7.0/(2.4 + LVESD)] × LVESD^3^; LVEDV (mL) = [7.0/(2.4 + LVEDD)] × LVEDD^3^; and LV mass (g) = 0.8 × {1.04 [(LVEDD + SWT + LVPW)^3^ − LVEDD^3^]} + 0.6, where LVEDD, SWT and LVPW are measured in cm; and LVEF (%) = [(LVEDV – LVESV)/LVEDV] × 100.

Diastolic function was assessed by measuring the transmitral flow rate (pulsed-wave Doppler, apical four-chamber view) and determining *E* and *A* wave velocities (both in cm/s).

Relative wall thickness (RWT) was calculated with the formula RWT = (SWT + LVPW)/(LVEDD), which allowed grouping the athletes into four categories [14]:

- Normal geometry, RWT ≤ 0.42 cm and LV mass/BSA  ≤116 (males) or ≤ 96 g/m^2^ (females)
- Concentric remodeling, RWT > 0.42 cm and LV mass/BSA ≤ 116 (or 96) g/m^2^
- Concentric hypertrophy, RWT > 0.42 cm and LV mass/BSA > 116 (or 96) g/m^2^
- Eccentric hypertrophy, RWT ≤ 0.42 cm and LV mass/BSA > 116 (or 96) g/m^2^

LV dimensions were expressed relative to BSA (in m^2^, calculated as 0.007184 × height (cm)^0.725^ × weight (kg)^0.425^ [15]). We also assessed the number of athletes with LV dimensions (thickness and cavity) above those considered ‘normal’ for the general population [14].

In addition, all participants underwent a cardiopulmonary exercise test until volitional exhaustion to determine VO_2max_ using a breath-by-breath metabolic cart (Jaeger Oxycon Pro System; Jaeger, Wuerzburg, Germany), as detailed elsewhere [13, 16]. Depending on the athlete’s sports discipline, the test was performed on a treadmill, cycle-ergometer, or rowing-ergometer.

### App “Online Calculator”

We developed a web application using Google Sheets as a database, Javascript for statistical calculations, and HTML 5 for presentation. The data of the athletes that are entered into the application user interface are statistically evaluated against (but not stored in) our reference database.

### Statistical Analyses

Data are shown as mean (standard deviation (SD)), and the 95th percentile (P95) is also shown for each variable, as a measure of the upper limit of normality. The χ^2^ test (or Fisher’s exact test if > 20% of the cells in the cross-table had an expected frequency < 5) was used to compare the proportion of the four types of cardiac geometry between the two sexes and also attending to the dynamic/static component of each sport. Unpaired Student’s t test and one-way analysis of variance (ANOVA) were used for comparisons of LV dimensions for sex and sport category, respectively, with the Bonferroni test used post hoc for pairwise comparisons when a significant group (*i.e.*, sex or sports category) effect was found. Effect size was determined with partial eta squared (*η*^2^_*p*_, for comparisons of geometry proportions) and Cohen’s d (for comparisons of the different LV dimensions) and was considered small (*η*^2^_*p*_ ≥ 0.01 or *d* ≥ 0.2), medium (*η*^2^_*p*_ ≥ 0.06 or *d* ≥ 0.5) or large (*η*^2^_*p*_ ≥ 0.14 or *d* ≥ 0.8) [17]. We also determined Pearson’s correlation coefficients between VO_2max_ and the different LV dimensions. Finally, for the cardiac dimension variables shown in the App online calculator, we reported Z-scores (*i.e.*, an indicator of how far [that is, how many SD above or below] from the population mean [μ] a data point [X] is), where *Z* = (*x* – *μ*)/SD. Statistical analyses were performed with Statistical Package for Social Sciences (SPSS) (IBM, Armonk, NY).

## Results

**Fig. 1:**
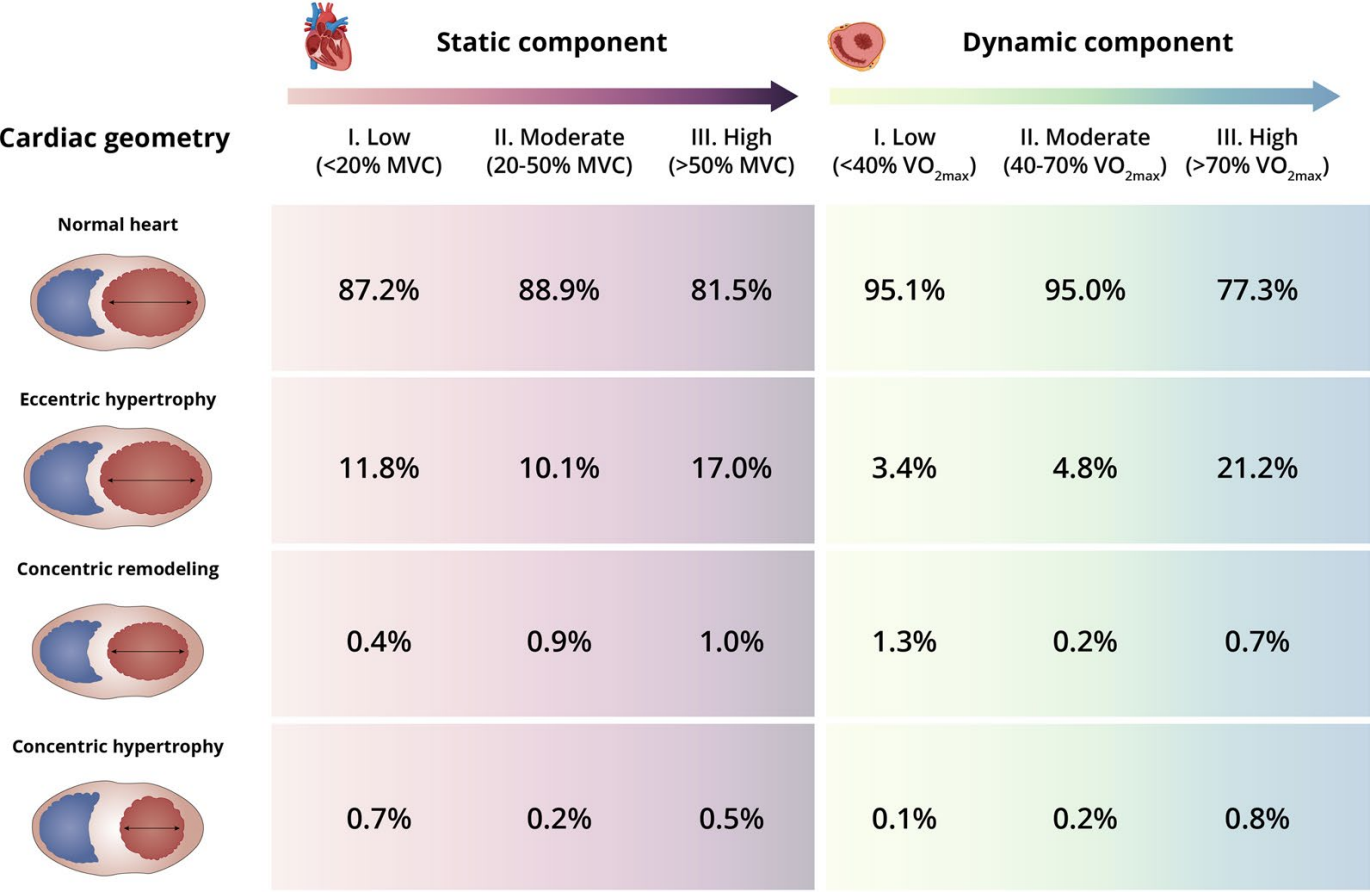
Prevalence of cardiac geometries in elite athletes (both sexes combined) attending to the static and dynamic component of their sport. Abbreviations: VO_2max_, maximum oxygen uptake; MVC, maximal voluntary contraction

We evaluated 4596 consecutive athletes. All subjects had been participating in high competition level (*i.e.*, within the national team and participating in international events) for 1 to 22 years. The proportion of those with ≤ 1-year experience at such high competition level was essentially minor for all sport categories, especially in male athletes (≤ 4.8%, vs. ≤ 10.7% in female athletes) (Additional file 1). A total of 3282 white elite athletes from 76 different specialties of 42 sport disciplines (37.8% women) met all inclusion criteria and were thus studied. Most data for the present study (~ 85%) were gathered during the preparatory macrocycle for the sport in question. The main demographic and body dimension characteristics for male and female athletes, respectively, were as follows: mean ± SD age, 24.1 ± 5.8 years (range [minimum to maximum individual value] 18–53) and 21.5 ± 5.0 years (16–43) years; height, 179.9 ± 9.3 cm (150.2–222.2) and 167.1 ± 7.9 cm (141.0–196.7); weight, 76.5 ± 13.6 kg (47.8–142.3) and 60.8 ± 10.5 kg (33.8–130.3); and BSA 1.96 ± 0.20 m^2^ (1.43–2.92) and 1.68 ± 0.16 m^2^ (1.18–2.36). Except for male athletes participating in sports with a low (or moderate) dynamic component, the proportion of participants aged > 35 years was low (≤ 4.1% and ≤ 5.3% in male and female athletes, respectively) (Additional file 1). Training volume averaged 19 ± 9 h/week and 19 ± 10 h/week, respectively, and elite competition experience was 9 ± 5 years and 8 ± 4 years. Mean VO_2max_ was 57.2 ± 9.1 mL/kg/min and 48.3 ± 7.7 mL/kg/min, whereas systolic/diastolic blood pressure averaged 121 ± 10 mmHg and 113 ± 10 mmHg/67 ± 7 mmHg and 63 ± 7 mmHg. The majority of athletes (85.4% of total) showed normal cardiac geometry (Fig. 1), particularly female athletes (Table 1). The second most prevalent pattern (13.4%) was eccentric hypertrophy, which was more frequent in males than in females, and concentric remodeling and hypertrophy were very uncommon (prevalence for each condition consistently ≤ 0.9% in both sexes). With the exception of LVEDD/BSA, male athletes had higher mean values than female athletes for almost all actual or BSA-corrected cardiac dimensions.

**Table 1:** Cardiac geometry and left ventricular measures by sex in athletes (all sports combined)

	Men (*n* = 2041)	Women (*n* = 1241)	*p* value	Effect size*
Cardiac geometry	Prevalence		< 0.001	0.015
Normal (%)	82.0	90.9		
Eccentric hypertrophy (%)	16.5	8.3		
Concentric remodeling (%)	0.9	0.5		
Concentric hypertophy (%)	0.6	0.3		

LV measures	Mean	P95	Mean	P95		
LVEF (%)	61 (7)	**72**	61 (7)	**72**	0.036	0.072
SWT (mm)	9 (1)	**11**	8 (1)	**9**	< 0.001	1.370
SWT / BSA (mm/m^2^)	4.7 (0.6)	**5.8**	4.6 (0.6)	**5.6**	0.002	0.167
LVEDD (mm)	55 (4)	**63**	49 (4)	**56**	< 0.001	1.409
LVEDD / BSA (mm/m^2^)	28 (3)	**33**	30 (3)	**34**	< 0.001	0.380
LVPW (mm)	9 (1)	**11**	8 (1)	**9**	< 0.001	1.313
LVPW / BSA (mm/m^2^)	4.6 (0.6)	**5.6**	4.5 (0.6)	**5.5**	0.003	0.167
LVEDV (mL)	150 (28)	**198**	115 (21)	**151**	< 0.001	1.370
LVEDV / BSA (mL/m^2^)	77 (13)	**100**	69 (11)	**87**	< 0.001	0.679
LV mass (g)	190 (43)	**271**	126 (29)	**177**	< 0.001	1.675
LV mass / BSA (g/m^2^)	97 (20)	**133**	75 (15)	**102**	< 0.001	1.855
*E* wave (cm/s)	85 (14)	**109**	92 (14)	**116**	< 0.001	0.461
*A* wave (cm/s)	43 (11)	**61**	45 (13)	**63**	< 0.001	0.147

Data of LV measures are mean (SD) and 95th (P95) percentile (in bold)

*BSA* body surface area, *LVEDD* left ventricular end-diastolic diameter, *LVEDV* left ventricular end-diastolic volume, *LVEF* left ventricular ejection fraction, *LVPW* LV posterior wall, *SWT* septal wall thickness

*Effect size assessed with partial eta squared (for between-sex comparisons of geometry proportions with the χ^2^ test [upper part of the Table]) and Cohen’s D (for between-sex comparisons of the different LV dimensions with the Student’s t test [lower part of the Table])

In both male (Additional file 2) and female (Additional file 3) athletes, the proportion of normal cardiac geometry and eccentric hypertrophy decreased and increased, respectively, with the dynamic component of the sport irrespective of the static component. Mean and P95 values of VO_2max_ and cardiac dimensions also increased in both sexes with the dynamic component of the sport, with the highest values found for those with a high (> 70% VO_2max_) component. The proportion of normal cardiac geometry and eccentric hypertrophy also showed a significant decrease and increase, respectively, with the static component of the sport in male (Additional file 4) but not in female (Additional file 5) athletes. Although the trend was less pronounced when compared with the dynamic component, mean cardiac dimensions (especially when BSA-corrected) also increased overall with the static component of the sport despite an opposite trend for VO_2max_.

**Fig. 2:**
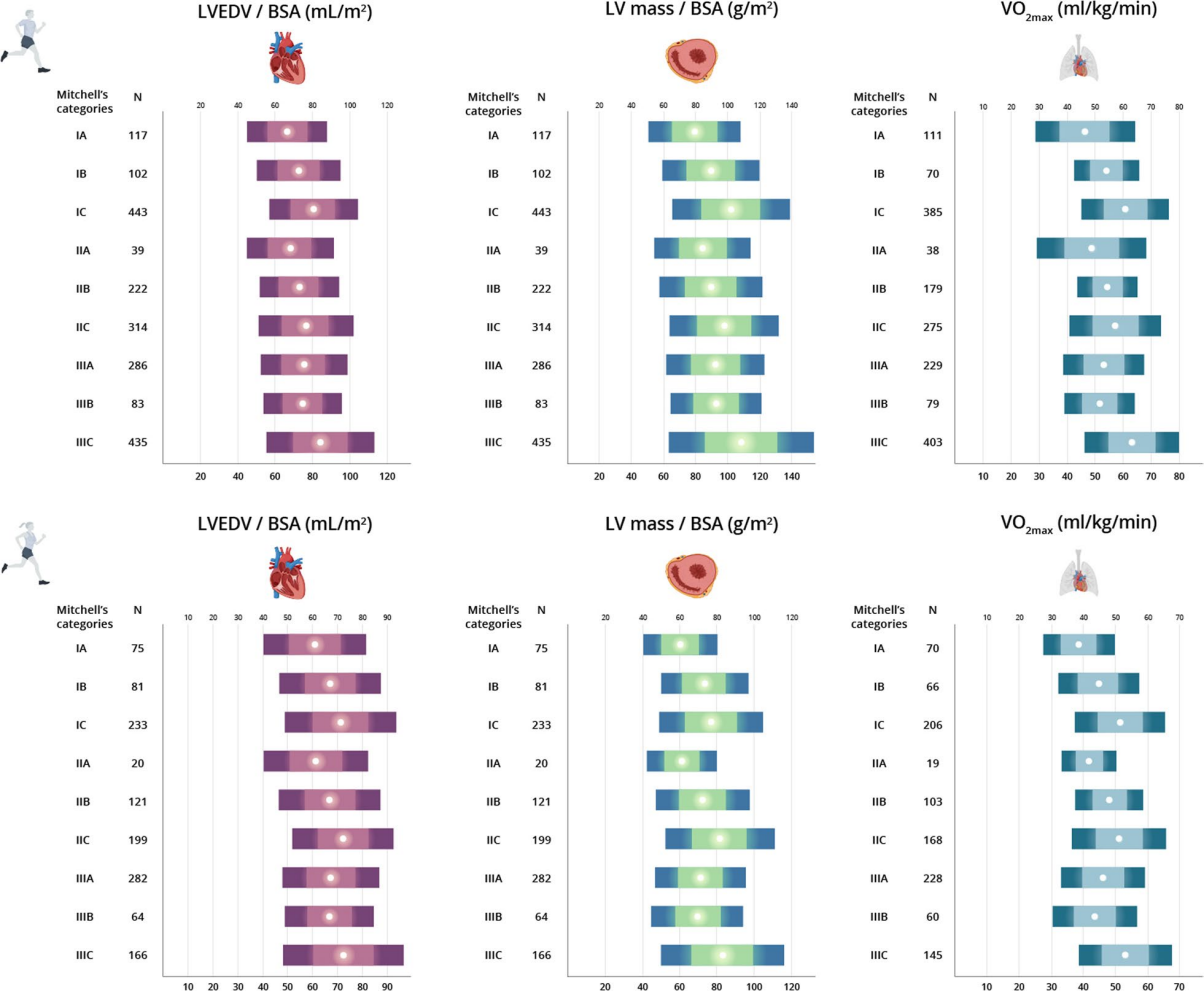
Left ventricular end-diastolic volume (LVEDV), LV mass and maximum oxygen uptake (VO_2max)_ for each Mitchell’s category by sex. LVEDV and LV mass are expressed relative to body surface area (BSA) in male (upper panel) and female (lower panel) athletes. Data are mean (circles) ± 1SD and ± 2SD (lighter and darker color horizontal bars, respectively)

**Fig. 3:**
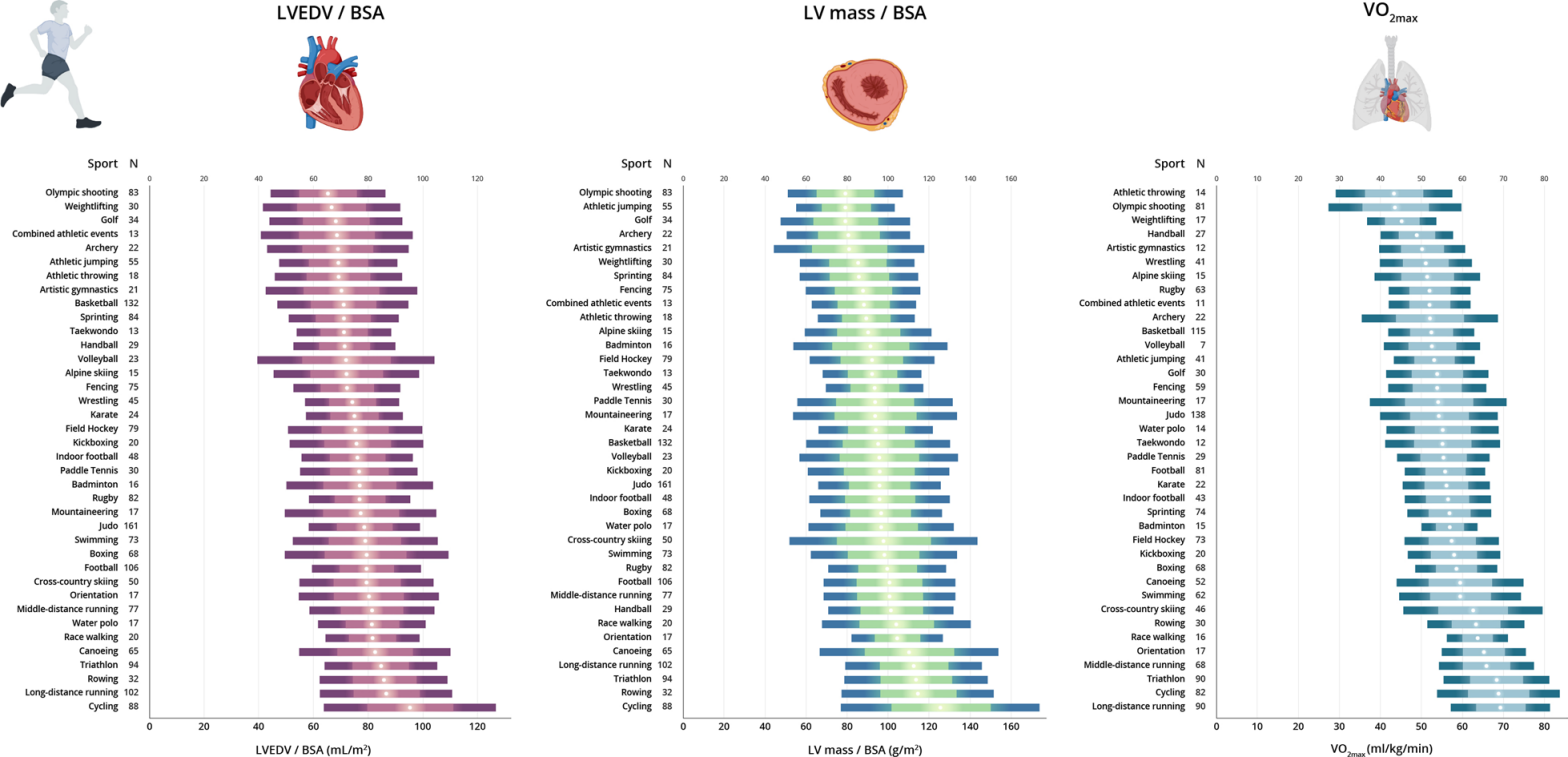
Left ventricular end-diastolic volume (LVEDV), LV mass and maximum oxygen uptake (VO_2max)_ for each main individual sport in male athletes. LVEDV and LV mass are expressed relative to body surface area (BSA). Data are mean (circles) ± 1SD and ± 2SD (lighter and darker color horizontal bars, respectively)

**Fig. 4:**
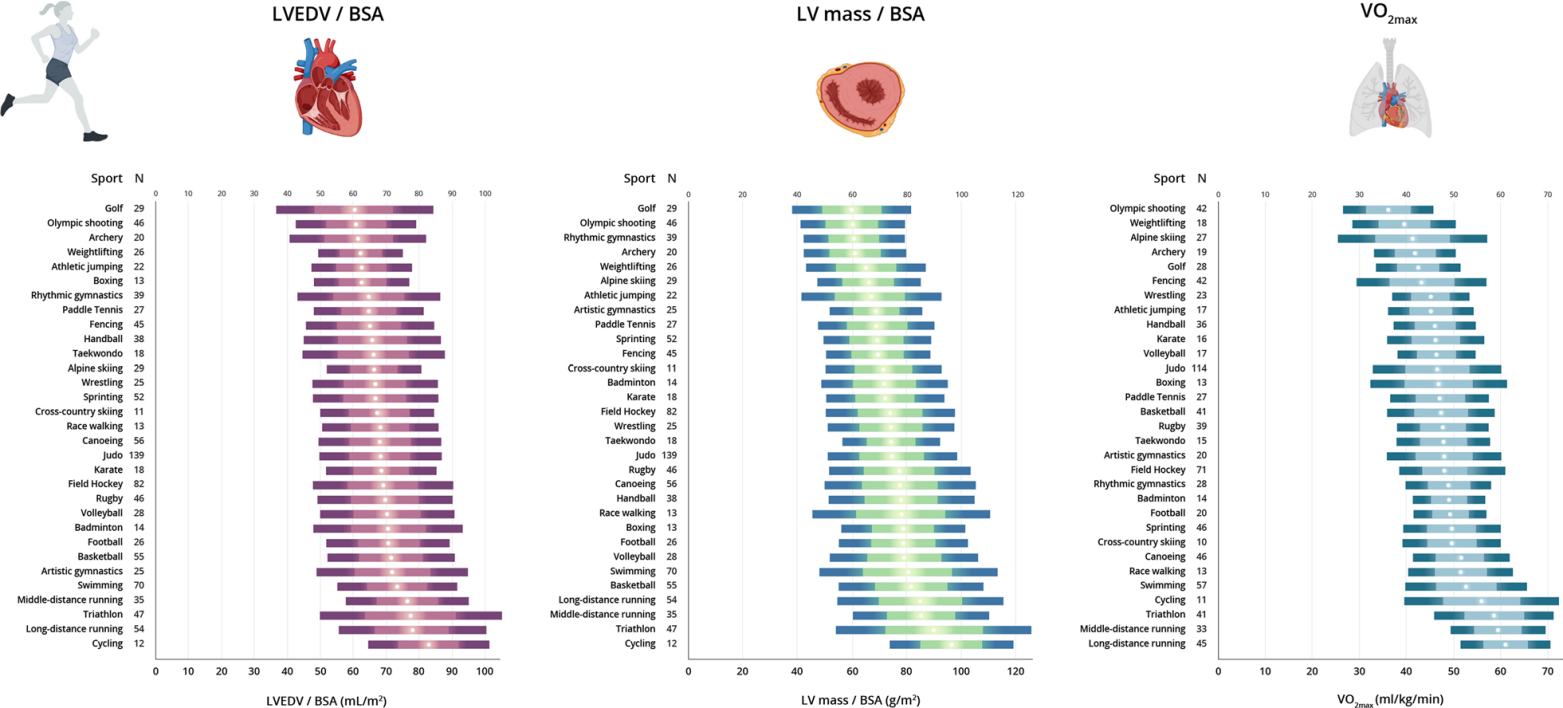
Left ventricular end-diastolic volume (LVEDV), LV mass and maximum oxygen uptake (VO_2max)_ for each main individual sport in female athletes. LVEDV and LV mass are expressed relative to body surface area (BSA). Data are mean (circles) ± 1SD and ± 2SD (lighter and darker color horizontal bars, respectively)

Normative values of LV dimensions in male and female athletes attending to the main sport categories are shown in Tables 2 and 3, respectively. The P95 values did not exceed the following limits in any of the sport categories for male and female athletes, respectively: SWT, 12 mm and 10 mm; LVPW, 11 mm and 10 mm; and LVEDD, 64 mm and 57 mm. Values of BSA-indexed LVEDV, LV mass and VO_2max_ for each sport category by sex are shown in Fig. 2 (see also Figs. 3, 4 for the values of each individual sport in male and female athletes, respectively).

**Table 2:** Normative values of left ventricular measures for male athletes attending to sport category

	Low static						Moderate static						High static					
	Low dynamic		Moderate dynamic		High dynamic		Low dynamic		Moderate dynamic		High dynamic		Low dynamic		Moderate dynamic		High dynamic	
	IA (*n* = 117)		IB (*n* = 102)		IC (*n* = 443)		IIA (*n* = 39)		IIB (*n* = 222)		IIC (*n* = 314)		IIIA (*n* = 286)		IIIB (*n* = 83)		IIIC (*n* = 435)	
	Mean	P95	Mean	P95	Mean	P95	Mean	P95	Mean	P95	Mean	P95	Mean	P95	Mean	P95	Mean	P95
VO_2max_ (mL/kg/min)	46.3 (8.8)	**60.3**	54.0 (5.7)	**64.2**	60.7 (7.7)	**73.9**	48.9 (9.6)	**65.7**	54.4 (5.4)	**63.3**	56.8 (8.3)	**71.2**	53.1 (7.3)	**64.0**	51.8 (6.2)	**62.5**	63.1 (8.2)	**76.5**
LVEF (%)	61 (7)	**72**	59 (7)	**73**	61 (7)	**71**	62.4 (6.3)	**72**	61 (7)	**72**	60 (7)	**72**	60 (7)	**71**	61 (8)	**74**	61 (67)	**73**
SWT (mm)	9 (1)	**11**	9 (1)	**11**	9 (1)	**11**	9 (1)	**11**	9 (1)	**11**	10 (1)	**11**	9 (1)	**11**	9 (1)	**10**	9 (1)	**12**
SWT / BSA (mm/m^2^)	4.4 (0.5)	**5.4**	4.5 (0.5)	**5.5**	4.9 (0.7)	**6.1**	4.6 (0.6)	**5.8**	4.5 (0.5)	**5.4**	4.5 (0.6)	**5.6**	4.6 (0.6)	**5.5**	4.7 (0.6)	**5.4**	5.0 (0.7)	**6.0**
LVEDD (mm)	52 (4)	**57**	55 (4)	**60**	55 (4)	**61**	52 (4)	**60**	55 (4)	**62**	57 (5)	**64**	55 (5)	**61**	54 (4)	**63**	57 (5)	**64**
LVEDD /BSA (mm/m^2^)	27 (2)	**31**	27 (2)	**32**	30 (2)	**34**	27 (3)	**31**	27 (2)	**30**	27 (3)	**33**	28 (3)	**32**	28 (2)	**32**	30 (3)	**35**
LVPW (mm)	8 (1)	**10**	9 (1)	**11**	9 (1)	**11**	8 (1)	**11**	9 (1)	**10**	9 (1)	**11**	9 (1)	**10**	9 (1)	**10**	9 (1)	**11**
LVPW/BSA (mm/m^2^)	4.3 (0.5)	**5.3**	4.4 (0.5)	**5.2**	4.8 (0.6)	**5.8**	4.4 (0.5)	**5.8**	4.3 (0.5)	**5.2**	4.4 (0.6)	**5.3**	4.5 (0.5)	**5.4**	4.6 (0.5)	**5.6**	4.8 (0.6)	**5.9**
LVEDV (mL)	128 (21)	**162**	146 (26)	**183**	151 (23)	**187**	131 (24)	**178**	146 (26)	**194**	160 (30)	**209**	146 (27)	**189**	143 (23)	**199**	159 (29)	**210**
LVEDV/BSA (mL/m^2^)	66 (11)	**84**	73 (11)	**94**	80 (12)	**102**	68 (12)	**85**	73 (11)	**89**	76 (12)	**98**	75 (11)	**93**	74 (11)	**98**	84 (15)	**111**
LV mass (g)	154 (30)	**207**	180 (38)	**259**	191 (35)	**247**	162 (31)	**227**	180 (39)	**252**	208 (46)	**297**	180 (40)	**256**	178 (34)	**257**	206 (46)	**291**
LV mass / BSA (g/m^2^)	79 (14)	**104**	90 (15)	**118**	102 (18)	**135**	84 (15)	**107**	89 (16)	**114**	98 (17)	**125**	92 (15)	**116**	93 (14)	**119**	109 (22)	**147**

Data are mean (SD) and 95th (P95) percentile (in bold)

*BSA* body surface area, *LVEDD* left ventricular end-diastolic diameter, *LVEDV* left ventricular end-diastolic volume, *LVEF* left ventricular ejection fraction, *LVPW* LV posterior wall, *SWT* septal wall thickness, VO_2max_, maximum oxygen uptake

**Table 3:** Normative values of left ventricular measures for female athletes attending to sport category

	Low static						Moderate static						High static					
	Low dynamic		Moderate dynamic		High dynamic		Low dynamic		Moderate dynamic		High dynamic		Low dynamic		Moderate dynamic		High dynamic	
	IA (*n* = 75)		IB (*n* = 81)		IC (*n* = 233)		IIA (*n* = 20)		IIB (*n* = 121)		IIC (*n* = 199)		IIIA (*n* = 282)		IIIB (*n* = 64)		IIIC (*n* = 166)	
	Mean	P95	Mean	P95	Mean	P95	Mean	P95	Mean	P95	Mean	P95	Mean	P95	Mean	P95	Mean	P95
VO_2max_ (mL/kg/min)	38.7 (5.5)	**47.5**	44.6 (6.3)	**53.8**	51.4 (7.0)	**65.1**	41.8 (4.2)	**–**	48.5 (5.0)	**58.0**	51.4 (7.2)	**65.4**	46.1 (6.5)	**57.7**	43.5 (6.6)	**52.8**	53.1 (7.3)	**66.3**
LVEF (%)	62 (8)	**72**	60 (7)	**71**	61 (8)	**73**	62 (8)	**–**	62 (6)	**70**	61 (7)	**74**	61 (7)	**72**	60 (6)	**71**	61 (7)	**71**
SWT (mm)	7 (1)	**8**	8 (1)	**9**	8 (1)	**9**	7 (1)	**–**	7 (1)	**9**	8 (1)	**10**	7 (1)	**9**	7 (1)	**9**	7 (1)	**9**
SWT / BSA (mm/m^2^)	4.2 (0.5)	**5.2**	4.4 (0.4)	**5.1**	4.7 (0.6)	**5.8**	4.2 (0.4)	**–**	4.5 (0.5)	**5.5**	4.5 (0.5)	**5.6**	4.6 (0.6)	**5.5**	4.5 (0.6)	**5.6**	4.8 (0.6)	**5.5**
LVEDD (mm)	46 (4)	**53**	50 (4)	**57**	49 (3)	**55**	47 (4)	**–**	49 (3)	**56**	51 (4)	**57**	48 (4)	**55**	48 (3)	**54**	50 (4)	**57**
LVEDD /BSA (mm/m^2^)	28 (2)	**32**	28 (2)	**32**	30 (3)	**36**	28 (2)	**–**	29 (2)	**32**	29 (2)	**34**	30 (3)	**35**	30 (2)	**33**	30 (3)	**35**
LVPW (mm)	7 (1)	**8**	8 (1)	**9**	8 (1)	**9**	7 (1)	**–**	8 (1)	**9**	8 (1)	**10**	8 (1)	**9**	7 (1)	**9**	8 (1)	**9**
LVPW/BSA (mm/m^2^)	4.1 (0.5)	**5.0**	4.3 (0.4)	**5.1**	4.7 (0.6)	**5.6**	4.1 (0.4)	**–**	4.4 (0.5)	**5.2**	4.6 (0.6)	**5.4**	4.5 (0.5)	**5.6**	4.5 (0.6)	**5.4**	4.8 (0.6)	**5.3**
LVEDV (mL)	101 (21)	**136**	119 (22)	**162**	115 (18)	**149**	104 (18)	**–**	114 (19)	**154**	128 (22)	**160**	110 (20)	**150**	109 (16)	**143**	120 (21)	**157**
LVEDV/BSA (mL/m^2^)	61 (10)	**80**	67 (10)	**83**	71 (11)	**90**	61 (10)	**–**	67 (10)	**85**	72 (10)	**87**	67 (10)	**85**	67 (9)	**84**	72 (12)	**92**
LV mass (g)	100 (19)	**132**	130 (28)	**191**	124 (23)	**167**	104 (19)	**–**	123 (24)	**176**	144 (32)	**210**	117 (28)	**173**	114 (22)	**155**	136 (30)	**197**
LV mass / BSA (g/m^2^)	60 (10)	**77**	73 (12)	**96**	77 (14)	**106**	61 (9)	**–**	72 (12)	**100**	81 (14)	**109**	71 (12)	**92**	70 (12)	**92**	83 (16)	**117**

Data are mean (SD) and 95th (P95) percentile (in bold)

*BSA* body surface area, *LVEDD* left ventricular end-diastolic diameter, *LVEDV* left ventricular end-diastolic volume, *LVEF* left ventricular ejection fraction, *LVPW* posterior wall, *SWT* septal wall thickness, *VO*_*2max*_ maximum oxygen uptake

The online and app-based calculator (https://sites.google.com/lapolart.es/athlete-lv/welcome?authuser=0) allows the determination of sport- and Mitchell-category-specific Z-scores for LV geometry, SWT, LVPW, LV mass/BSA, RWT, and LVEDD and the corresponding percentile value for each of the LV measures in a given healthy adult athlete.

**Fig. 5:**
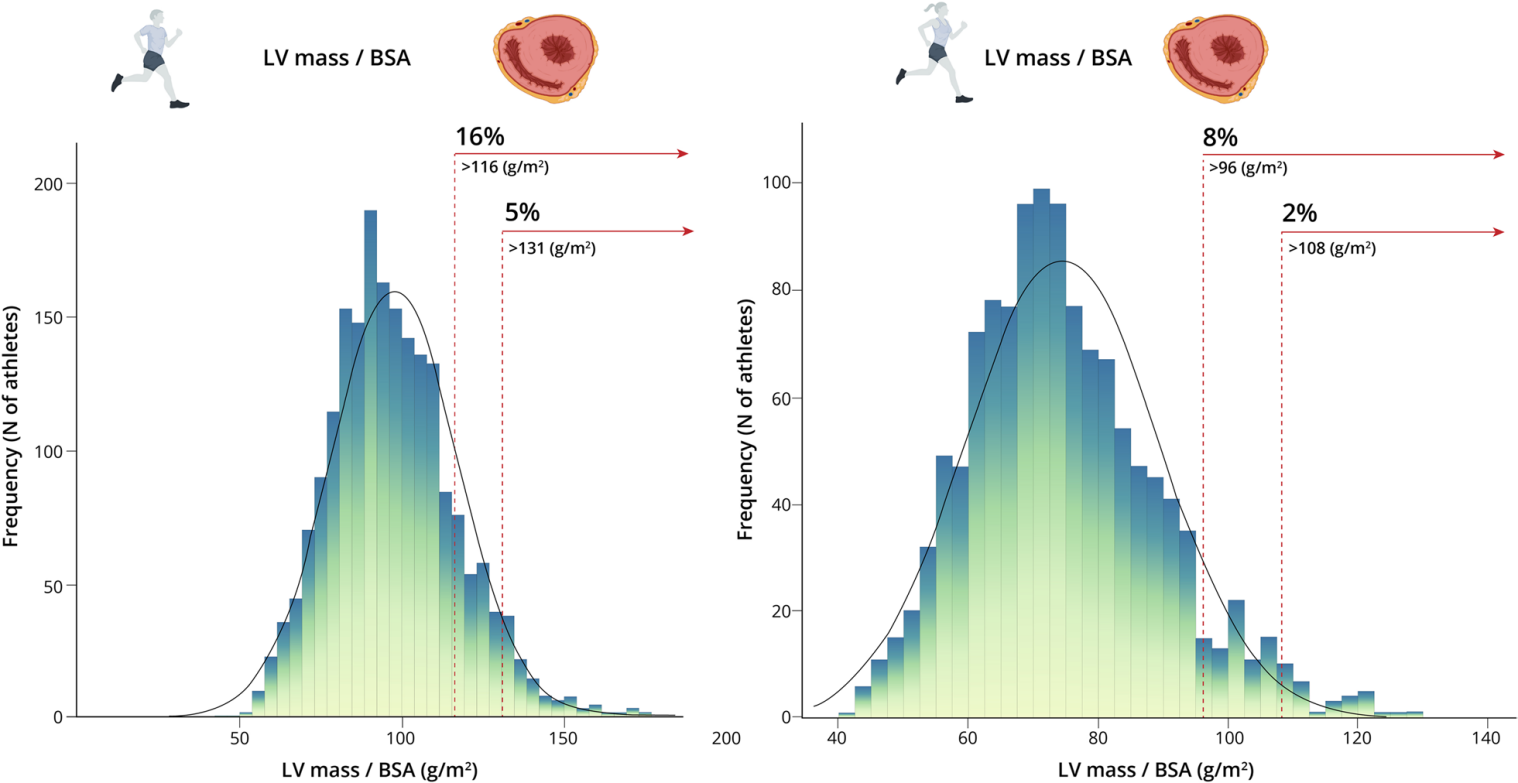
Frequency of different levels of left ventricular (LV) mass expressed relative to body surface area (BSA) in male (panel A) and female athletes (panel B). The prevalence of athletes with values suggestive of LV hypertrophy (*i.e.*, LV mass  >116 g or LV mass/BSA  >131 g/m^2^ for male athletes and LV mass > 96 g or LV mass/BSA > 108 g/m^2^ for female athletes, respectively (14)) is shown

As for ‘abnormal’ individual values, a very low proportion of male (*n* = 5, 0.24%) and female (*n* = 2, 0.16%) athletes had SWT values > 13 mm and > 11 mm, respectively, with SWT values between 12 and 13 mm found in 1.3% (*n* = 26) of male athletes. No male or female athlete had LVPW values > 13 mm or > 11 mm, respectively, with seven male athletes (0.3%) presenting values between 12 and 13 mm. Five hundred and fifty-nine (27%) and 280 female (23%) athletes had LVEDD values > 58 mm and > 52 mm, respectively, and 89 male (4%) and 62 (6%) female athletes had LVEDD values > 63 mm and > 56 mm, respectively. Four male athletes with LVEDD values > 63 mm had an abnormally low LVEF (*i.e.*, below the 52% limit for elite athletes[13]) but none had a diagnosed cardiac pathology. By contrast, no female athlete with a LVEDD > 56 mm had an LVEF below 52%. 17.1% and 8.6% of the male and female athletes, respectively, had a LV mass/BSA > 115 and > 95 (Fig. 5).

Finally, except for LVEF, VO_2_max was significantly correlated with all the LV measures we studied in both sexes, although correlations were weak (*i.e.*, Pearson’s r-coefficient ≥ 0.5 only for LVEDD/BSA in men) (Additional file 6).

## Discussion

The present study describes the LV dimensions in a large cohort of white elite athletes—to our knowledge, the largest one assessed to date—and determines how sex and sport type (with the corresponding changes in static and dynamic component) are associated with different LV characteristics. Our main finding is that most LV measures in elite white athletes (*i.e.*, overall aged < 30 years [~ 20 to 25 years on average] and with a mean competition experience of ~ 9 years) are within the established limits for the general population; however, some LV dimensions are above these limits in a non-negligible proportion of athletes.

The great majority of athletes in this study (~ 85% of total, both sexes combined) had normal LV geometry. Yet, in line with previous research [18], eccentric hypertrophy was relatively prevalent in both male and female athletes and was the second most common pattern (13.4% of total). By contrast, concentric remodeling or hypertrophy was present in less than 1% of the cohort in total, supporting the notion that cardiac remodeling characterized by increases in LV wall thickness with no proportional changes in cavity dimensions is less common among competitive athletes than previously thought [3]. The very low prevalence (0.5% of total) of concentric LV hypertrophy (as determined by RWT and LV mass index) among elite athletes is overall in line with a classic study by Pelliccia et al*.* [7], in which 1.7% of 947 athletes presented with this condition. Contrastingly, Basavarajaiah et al. [6] found a higher prevalence of LV concentric hypertrophy, particularly among black male athletes (18% vs. 4% in white male athletes). The lack of agreement between studies might be explained by differences in measuring techniques (*i.e.*, parasternal long-axis view in the present study vs. the short-axis view in the Basavarajaiah et al*.* study [6]), or in some cohort characteristics such as race, competitive experience or type of sport involved.

Female athletes had a lower LV mass than males, as well as a greater prevalence of normal LV geometry. These findings point to a sex-specific pattern of exercise-induced cardiac remodeling among elite athletes, which is consistent with the previous studies showing that female athletes seemed to have a different pattern of remodeling than their male counterparts, including higher BSA-indexed dimensions (which in our cohort was only true, however, for LVEDD/BSA), lower values of LV wall thickness and LV mass, and a higher prevalence of normal LV geometry [19, 20]. On the other hand, it must be noted that the normative values we reported are not necessarily applicable to older athletes. For instance, a recent study in master athletes (36–83 years) showed cardiac remodeling to be shifted toward normal geometry in sprinters and toward concentric remodeling and hypertrophy in endurance runners [21].

Regarding the type of sport, the highest actual and BSA-indexed values of LV thickness (SWT or LVPW) and mass were found for sports with a greater dynamic (‘endurance’) component. Although the trend was less marked than for the dynamic component, the aforementioned variables (at least when BSA-corrected) also increased with the static (‘resistance’) component of the sport in male athletes. Importantly, these findings are overall at odds with the Morganroth hypothesis of a dichotomous LV remodeling pattern in endurance and resistance athletes (*i.e.*, increased LV mass mostly due to an increased LVEDV in the former, or to an increased SWT and LVPW in the latter) [3, 22]. In fact, the endurance athletes in our cohort had greater values for both LV wall thickness (SWT or LVPW) and LV mass than the resistance athletes. This might be explained by the fact that the Morganroth hypothesis does not consider the increase in intrathoracic pressure during isometric exercise as an important LV wall stress determinant that would compensate for increased intraventricular pressure [23]. Indeed, recent prospective and cross-sectional studies seem to point toward a lesser-than-expected increase in LV wall thickness in sports with a high isometric component [18, 19, 24].

There is common agreement that regular endurance training results in cardiac chamber enlargement; however, studies in endurance athletes show that LV dimensions are usually within normal ranges [2, 10, 25, 26]. Similarly, in the present study, we found that the mean values for LVEDD in both sexes were within normal limits for the general population [14] (≤ 58 mm [males] and ≤ 52 mm [females]), even for those sports with the highest dynamic component. That being said, a substantial proportion of athletes (27% [male] and 23% [female]) had individual LVEDD values above the aforementioned upper limits. In addition, the P95 value for LVEDD in IIIC sports (with both high dynamic and static components, such as cycling, triathlon, rowing or canoeing/kayaking) was 64 mm and 57 mm for male and female athletes, respectively. A classic study by Pelliccia et al*.* [10] found overall higher LVEDD values (*i.e.*, 14% had a LVEDD > 60 mm). The differences between their study and ours might be attributable to the lower proportion of female athletes in the former (27%, vs. ~ 38% here).

Some limitations of the present study should be acknowledged, such as the uneven representation of athletes in the different sports categories, with some underrepresented (*e.g.*, IIA), particularly in female athletes. The use of the classic Mitchell’s classification for sports categorization might also be viewed as a potential limitation. In this regard, we believe there is no unanimity as to the best means of categorizing the different sports based on hemodynamic and overall physiological loads, especially at the highest competition level, which was the case of our cohort. More recent alternatives to the Mitchell’s classification are available, notably those recently proposed by the 2020 European Society of Cardiology guidelines on sports cardiology and exercise in patients with cardiovascular disease [27]. In this reference document, sporting disciplines were classified in relation to the predominant component (skill, power, mixed, and endurance) and intensity of exercise (low, medium, and high). In this effect, we believe that any classification can have both advantages and disadvantages given the difficulty of categorizing a given sport taking into account both competitions and training hours. Indeed, heterogeneity is to be expected not only within a given sport (*e.g.*, in soccer, midfielders or wingbacks are exposed to much higher dynamic loads and exercise intensities than goalkeepers despite both enrolled in the same sport) but also within the same athlete over his/her career (*e.g.*, due to changes in training toward more endurance-oriented workouts [in the case of a former middle-distance runner moving to longer distances] or power/strength sessions in endurance athletes [to prevent injuries or improve spring ability]). To further complicate the issue, all athletes spend much more time in training than in competition, and thus numerous different modes of exercise skills, modalities and intensities are likely to be involved to a lesser or a higher degree in weekly workouts irrespective of the essential nature of the main competition event in question. On the other hand, although the use of the 2D-guided M-mode approach used here to measure LV mass might not be the best option for assessing patients, here, we assessed healthy athletes and the M-method also has advantages (notably, being, subject to less measurement variability than the 2D-mode) [14], and in fact, there is evidence to support the accuracy of this method (*e.g.*, to predict cardiovascular outcomes from LV mass measures [28]).

Strengths of our study include the large number of elite athletes of both sexes participating in a wide variety of sports (all with measured VO_2max_), and the practical applicability of the normative values computed here, which can be easily used by clinicians to identify ‘at risk’ athletes (as well as to evaluate training adaptations over time at the cardiac structural level within a given sport) with the help of the online/mobile app we provide. The provision of cutoff values for LV dimensions according to sport practiced and sex should allow differentiation between normal (‘physiological’) and pathological cardiac remodeling in athletes, which could be useful in pre-participation screening and annual follow-ups.

## Conclusions

Most athletes (~ 85%) had normal LV geometry, with approximately one of eight athletes presenting with eccentric hypertrophy and only a minority showing concentric remodeling or hypertrophy (less than 1% for both conditions in either sex). The sex- and sport-specific normative values for LV dimensions provided here can serve to identify those athletes in which a detailed examination might be recommendable (*i.e.*, those with individual values ˃P95 for their sport, reflecting an abnormal cardiac adaptation).

## Supplementary Information

**Additional file 1:** Proportion of athletes with previous experience in the high competition ≤1 year and of athletes aged above 35 years, by sex and sport category.

**Additional file 2:** Cardiac geometry and left ventricular (LV) measures attending to the dynamic component of the sport in male athletes.

**Additional file 3:** Cardiac geometry and left ventricular (LV) measures attending to the dynamic component of the sport in female athletes.

**Additional file 4:** Cardiac geometry and left ventricular (LV) measures attending to the static component of the sport in male athletes.

**Additional file 5:** Cardiac geometry and left ventricular (LV) measures attending to the static component of the sport in female athletes.

**Additional file 6:** Pearson’s correlations between maximum oxygen uptake and left ventricular (LV) measures by sex (all sports combined).

## Data Availability

Data will be made available upon reasonable request to the corresponding author.

## Abbreviations

2D: Two-dimensional
*η*^2^_*p*_: Partial eta squared
ASE: American Society of Echocardiography
ANOVA: One-way analysis of variance
BSA: Body surface area
ECG: Electrocardiogram
LVEDD: Left ventricular end-diastolic diameter
LVEDV: Left ventricular end-diastolic volume
LVEF: Left ventricular ejection fraction
LVESD: Left ventricular end-systolic diameter
LVESV: Left ventricular end-systolic volume
LVPW: Left ventricular posterior wall thickness
MVC: Maximum voluntary contraction
P95: 95Th percentile
RWT: Relative wall thickness
SD: Standard deviation
SPSS: Statistical package for social sciences (SPSS)
SWT: Septal wall thickness
VO_2max_: Maximum oxygen uptake
