# Supplementary material for: Normative Values for Sport-Specific Left Ventricular Dimensions and Exercise-Induced Cardiac Remodeling in Elite Spanish Male and Female Athletes
Source: Sports Med Open. 2022 Sep 15;8:116. doi: 10.1186/s40798-022-00510-2 (PMC9478009; doi:10.1186/s40798-022-00510-2)
Supplement: Supplementary file 1 — Additional file 1: Proportion of athletes with previous experience in the high competition ≤1 year and of athletes aged above 35 years, by sex and sport category. [file 40798_2022_510_MOESM1_ESM.docx]

**Supplementary file 1**. Proportion of athletes with previous experience in the high competition ≤1 year and of athletes aged above 35 years, by sex and sport category

|  | Low static | | | Moderate static | | | High static | | | p-value for  Mitchell’s category |
| --- | --- | --- | --- | --- | --- | --- | --- | --- | --- | --- |
|  | **Low dynamic** | **Moderate dynamic** | **High dynamic** | **Low dynamic** | **Moderate dynamic** | **High dynamic** | **Low dynamic** | **Moderate dynamic** | **High dynamic** |  |
|  | **IA** | **IB** | **IC** | **IIA** | **IIB** | **IIC** | **IIIA** | **IIIB** | **IIIC** |  |
|  | **Proportion (%) of athletes with ≤1 year-experience in high competition** | | | | | | | | |  |
| Male | 2.6 | 2.0 | 0.9 | 2.6 | 1.4 | 2.2 | 2.5 | 4.8 | 3.2 | 0.217 |
| Female | 10.7 | 4.9 | 4.3 | 10.0 | 7.4 | 2.0 | 4.6 | 9.4 | 6.0 | 0.093 |
|  | **Proportion (%) of athletes aged >35 years** | | | | | | | | |  |
| Male | 14.5 | 2.0 | 4.1 | 23.1 | 0.9 | 1.6 | 1.1 | 13.3 | 3.5 | <0.01 |
| Female | 5.3 | 1.2 | 4.3 | 0.0 | 0.0 | 1.0 | 0.0 | 0.0 | 1.8 | <0.01 |
