# Supplementary material for: Normative Values for Sport-Specific Left Ventricular Dimensions and Exercise-Induced Cardiac Remodeling in Elite Spanish Male and Female Athletes
Source: Sports Med Open. 2022 Sep 15;8:116. doi: 10.1186/s40798-022-00510-2 (PMC9478009; doi:10.1186/s40798-022-00510-2)
Supplement: Supplementary file 2 — Additional file 2: Cardiac geometry and left ventricular (LV) measures attending to the dynamic component of the sport in male athletes. [file 40798_2022_510_MOESM2_ESM.docx]

.

**Supplementary file 2.** Cardiac geometry and left ventricular (LV) measures attending to the dynamic component of the sport in male athletes.

|  | **A**  **(low,**  **< 40% VO_2max_) n = 442** | | **B**  **(moderate,**  **40-70%VO_2max_) n = 407** | | **C**  **(high,**  **> 70% VO_2max_) n = n = 1,102** | | **p-value for**  **group effect** | **Effect size*** |
| --- | --- | --- | --- | --- | --- | --- | --- | --- |
|  |  |  |  | |  |  |  |  |
| **VO_2max_ (mL/kg/min)** | **Mean** | **P95** | **Mean P95** | | **Mean** | **P95** |  |  |
|  | 50.7 (8.5)^b,c^ | **62.8** | 53.7 (5.8)^a,c^ **63.2** | | 60.8 (8.4)^a,b^ | **74.7** | <0.001 | 0.019 |
| **Cardiac geometry** |  |  | **Prevalence** | |  |  | <0.001 | 0.074 |
| Normal (%) | 93.7 |  | 93.9 |  | 73.7 |  |  |  |
| Eccentric hypertrophy (%) | 4.3 |  | 5.7 |  | 24.7 |  |  |  |
| Concentric remodeling (%) | 1.8 |  | 0.3 |  | 0.8 |  |  |  |
| Concentric hypertophy (%) | 0.2 |  | 0.3 |  | 0.8 |  |  |  |
|  |  |  |  |  |  |  |  |  |
| **Cardiac dimensions** | **Mean** | **P95** | **Mean** | **P95** | Mean | **P95** |  |  |
| LVEF (%) | 61 (7) | **72** | 61 (7) | **73** | 61 (7) | **72** | 0.972 | 0.001 |
| SWT (mm) | 9 (1) ^c^ | **11** | 9 (1) ^c^ | **11** | 9 (1) ^a,b^ | **12** | <0.001 | 0.004 |
| SWT / BSA (mm/m^2^) | 4.6 (0.5) ^c^ | **5.5** | 4.5 (0.5) ^c^ | **5.5** | 4.9 (0.6) ^a,b^ | **6.0** | <0.001 | 0.054 |
| LVEDD (mm) | 54 (4) ^b,c^ | **61** | 54 (4) ^a,c^ | **61** | 56 (4) ^a,b^ | **63** | <0.001 | 0.012 |
| LVEDD /BSA (mm/m^2^) | 28 (3) ^c^ | **32** | 28 (2) ^c^ | **31** | 29 (3) ^a,b^ | **34** | <0.001 | 0.086 |
| LVPW (mm) | 9 (1) ^c^ | **10** | 9 (1) ^c^ | **10** | 9 (1) ^a,b^ | **11** | <0.001 | 0.015 |
| LVPW / BSA (mm/m^2^) | 4.4 (0.5) ^c^ | **5.4** | 4.4 (0.5) ^c^ | **5.3** | 4.7 (0.6) ^a,b^ | **5.8** | <0.001 | 0.026 |
| LVEDV (mL) | 140 (27) ^b,c^ | **186** | 145 (26) ^a,c^ | **190** | 156 (27) ^a,b^ | **204** | <0.001 | 0.013 |
| LVEDV/BSA (mL/m^2^) | 72 (12) ^c^ | **92** | 73 (11) ^c^ | **90** | 81 (13) ^a,b^ | **104** | <0.001 | 0.031 |
| LV mass (g) | 172 (39) ^b,c^ | **243** | 179 (38) ^a,c^ | **251** | 201 (43) ^a,b^ | **279** | <0.001 | 0.015 |
| LV mass / BSA (g/m^2^) | 88 (16) ^c^ | **114** | 90 (15) ^c^ | **117** | 103 (20) ^a,b^ | **139** | <0.001 | 0.026 |

Data of LV measures are mean (SD) and 95th (P95) percentile. Abbreviations: BSA, body surface area; SWT, septal wall thickness; LVEDD, left ventricular end diastolic diameter; LVEDV, left ventricular end diastolic volume; LVEF, left ventricular ejection fraction; LVPW, LV posterior wall. Symbols: ^a^ p<0.05 vs. A; ^b^ p<0.05 vs. B; ^c^ p<0.05 vs. C; * assessed with partial eta squared.
