# Supplementary material for: Normative Values for Sport-Specific Left Ventricular Dimensions and Exercise-Induced Cardiac Remodeling in Elite Spanish Male and Female Athletes
Source: Sports Med Open. 2022 Sep 15;8:116. doi: 10.1186/s40798-022-00510-2 (PMC9478009; doi:10.1186/s40798-022-00510-2)
Supplement: Supplementary file 3 — Additional file 3: Cardiac geometry and left ventricular (LV) measures attending to the dynamic component of the sport in female athletes. [file 40798_2022_510_MOESM3_ESM.docx]

**Supplementary file 3.** Cardiac geometry and left ventricular (LV) measures attending to the dynamic component of the sport in female athletes.

|  | **A**  **(low,**  **<40% VO_2max_)**  **n = 379** | | **B**  **(moderate,**  **40-70%VO_2max_)**  **n = 266** | | **C**  **(high,**  **> 70% VO_2max_)**  **n = 598** | | **p-value for**  **group effect** | **Effect size*** |
| --- | --- | --- | --- | --- | --- | --- | --- | --- |
|  |  |  |  | |  |  |  |  |
| **VO_2max_ (mL/kg/min)** | **Mean** | **P95** | **Mean P95** | | **Mean** | **P95** |  |  |
|  | 44.2(6.9) ^b,c^ | **54.7** | 46.1 (6.2) ^a,c^ **55.5** | | 51.9 (7.2) ^a,b^ | **65.2** | <0.001 | 0.013 |
| **Cardiac geometry** |  |  | **Prevalence** | |  |  | <0.001 | 0.049 |
| Normal (%) | 96.8 |  | 96.6 |  | 84.6 |  |  |  |
| Eccentric hypertrophy (%) | 2.4 |  | 3.4 |  | 14.2 |  |  |  |
| Concentric remodeling (%) | 0.8 |  | 0 |  | 0.5 |  |  |  |
| Concentric hypertophy (%) | 0 |  | 0 |  | 0.7 |  |  |  |
|  |  |  |  |  |  |  |  |  |
| **Cardiac dimensions** | **Mean** | **P95** | **Mean** | **P95** | Mean | **P95** |  |  |
| LVEF (%) | 61.3 (6.8) | **72** | 60.8 (6.3) | **71** | 61 (7) | **73** | 0.591 | 0.001 |
| SWT (mm) | 7 (1) ^b,c^ | **9** | 8 (1) ^a,c^ | **9** | 8 (1) ^a,b^ | **10** | <0.001 | 0.012 |
| SWT / BSA (mm/m^2^) | 4.5 (0.6) ^c^ | **5.5** | 4.5 (0.5) ^c^ | **5.4** | 4.7 (0.6) ^a,b^ | **5.8** | <0.001 | 0.011 |
| LVEDD (mm) | 48 (4) ^b,c^ | **55** | 49 (4) ^b,c^ | **56** | 50 (4) ^a,b^ | **57** | <0.001 | 0.029 |
| LVEDD /BSA (mm/m^2^) | 29 (3) ^c^ | **34** | 29 (2) ^c^ | **32** | 30 (3) ^a,b^ | **35** | <0.001 | 0.014 |
| LVPW (mm) | 7 (1) ^b,c^ | **9** | 7 (1) ^a,c^ | **9** | 8 (1) ^a,b^ | **10** | <0.001 | 0.033 |
| LVPW/BSA (mm/m^2^) | 4.4 (0.6) ^c^ | **5.3** | 4.4 (0.5) ^c^ | **5.3** | 4.6 (0.6) ^a,b^ | **5.7** | <0.001 | 0.010 |
| LVEDV (mL) | 108 (20) ^b,c^ | **146** | 114 (20) ^a,c^ | **150** | 121 (21) ^a,b^ | **158** | <0.001 | 0.029 |
| LVEDV/BSA (mL/m^2^) | 66 (10) ^c^ | **83** | 67 (10) ^c^ | **84** | 72 (11) ^a,b^ | **91** | <0.001 | 0.003 |
| LV mass (g) | 113 (27) ^b,c^ | **167** | 123 (25) ^a,c^ | **170** | 135 (30) ^a,b^ | **186** | <0.001 | 0.033 |
| LV mass / BSA (g/m^2^) | 68 (12) ^b,c^ | **89** | 72 (12) ^a,c^ | **93** | 80 (15) ^a,b^ | **107** | <0.001 | 0.010 |

Data of LV measures are mean (SD) and 95th (P95) percentile. Abbreviations: BSA, body surface area; SWT, septal wall thickness; LVEDD, left ventricular end diastolic diameter; LVEDV, left ventricular end diastolic volume; LVEF, left ventricular ejection fraction; LVPW, LV posterior wall. Symbols: ^a^ p<0.05 vs. A; ^b^ p<0.05 vs. B; ^c^ p<0.05 vs. C; * assessed with partial eta squared.
