# Supplementary material for: Normative Values for Sport-Specific Left Ventricular Dimensions and Exercise-Induced Cardiac Remodeling in Elite Spanish Male and Female Athletes
Source: Sports Med Open. 2022 Sep 15;8:116. doi: 10.1186/s40798-022-00510-2 (PMC9478009; doi:10.1186/s40798-022-00510-2)
Supplement: Supplementary file 4 — Additional file 4: Cardiac geometry and left ventricular (LV) measures attending to the static component of the sport in male athletes. [file 40798_2022_510_MOESM4_ESM.docx]

**Supplementary file 4.** Cardiac geometry and left ventricular (LV) measures attending to the static component of the sport in male athletes.

|  | **I**  **(low,**  **< 20% MVC) n = 662** | | **II**  **(moderate,**  **20-50% MVC) n = 575** | | **III**  **(high,**  **> 50% MVC) n = 804** | | **p-value for group effect** | **Effect size*** |
| --- | --- | --- | --- | --- | --- | --- | --- | --- |
|  |  |  |  | |  | |  |  |
| **VO_2max_ (mL/kg/min)** | **Mean** | **P95** | **Mean P95** | | **Mean** | **P95** |  |  |
|  | 57.0(9.6) ^b,c^ | **72.7** | 55.6 (7.8) ^a,c^ **69.6** | | 58.7 (9.3) ^a,b^ | **74.5** | 0.001 | 0.230 |
| **Cardiac geometry** |  |  | **Prevalence** | |  |  | 0.001 | 0.020 |
| Normal (%) | 84.1 |  | 87.8 |  | 76.2 |  |  |  |
| Eccentric hypertrophy (%) | 14.4 |  | 10.8 |  | 22.3 |  |  |  |
| Concentric remodeling (%) | 0.6 |  | 1.2 |  | 1.0 |  |  |  |
| Concentric hypertophy (%) | 0.9 |  | 0.2 |  | 0.6 |  |  |  |
|  |  |  |  |  |  |  |  |  |
| **Cardiac dimensions** | **Mean** | **P95** | **Mean** | **P95** | **Mean** | **P95** |  |  |
| LVEF (%) | 61 (17) | **72** | 61 (7) | **72** | 61 (7) | **72** | 0.904 | 0.001 |
| SWT (mm) | 9 (1) ^b,c^ | **11** | 9 (1) ^a^ | **11** | 9 (1)^a^ | **12** | 0.004 | 0.050 |
| SWT / BSA (mm/m^2^) | 4.8(0.7) ^b,c^ | **5.9** | 4.5 (0.6)^a,c^ | **5.6** | 4.8 (0.6) ^a,b^ | **6.0** | <0.001 | 0.062 |
| LVEDD (mm) | 55 (4) ^b,c^ | **61** | 56 (5) ^a^ | **63** | 56 (5) ^a^ | **64** | <0.001 | 0.065 |
| LVEDD /BSA (mm/m^2^) | 29 (3) ^b,c^ | **33** | 27 (3) ^a,c^ | **31** | 29 (3) ^a,b^ | **34** | <0.001 | 0.067 |
| LVPW (mm) | 9 (1) ^c^ | **11** | 9 (1) | **11** | 9 (1) ^a^ | **11** | 0.011 | 0.038 |
| LVPW/BSA (mm/m^2^) | 4.6 (0.6) ^b^ | **5.7** | 4.3 (0.5) ^a,c^ | **5.3** | 4.7 (0.6) ^b^ | **5.7** | <0.001 | 0.047 |
| LVEDV (mL) | 146 (25) ^b,c^ | **184** | 153 (29) ^a^ | **200** | 152 (29) ^a^ | **204** | <0.001 | 0.063 |
| LVEDV/BSA (mL/m^2^) | 77 (13) ^b,c^ | **98** | 74 (12) ^a,c^ | **95** | 80 (14) ^a,b^ | **105** | <0.001 | 0.090 |
| LV mass (g) | 183 (38) ^b,c^ | **242** | 194 (45) ^a^ | **273** | 194 (45) ^a^ | **280** | <0.001 | 0.087 |
| LV mass / BSA (g/m^2^) | 96 (19) ^c^ | **129** | 94 (17) ^c^ | **122** | 101 (21) ^a,b^ | **140** | <0.001 | 0.128 |

Data of LV measures are mean (SD) and 95th (P95) percentile. Abbreviations: BSA, body surface area; SWT, septal wall thickness; LVEDD, left ventricular end diastolic diameter; LVEDV, left ventricular end diastolic volume; LVEF, left ventricular ejection fraction; LVPW, LV posterior wall. Symbols: ^a^ p<0.05 vs. A; ^b^ p<0.05 vs. B; ^c^ p<0.05 vs. C; * assessed with partial eta squared.
