# Supplementary material for: Normative Values for Sport-Specific Left Ventricular Dimensions and Exercise-Induced Cardiac Remodeling in Elite Spanish Male and Female Athletes
Source: Sports Med Open. 2022 Sep 15;8:116. doi: 10.1186/s40798-022-00510-2 (PMC9478009; doi:10.1186/s40798-022-00510-2)
Supplement: Supplementary file 5 — Additional file 5: Cardiac geometry and left ventricular (LV) measures attending to the static component of the sport in female athletes. [file 40798_2022_510_MOESM5_ESM.docx]

**Supplementary file 5.** Cardiac geometry and left ventricular (LV) measures attending to the static component of the sport in female athletes.

|  | **I**  **(low,**  **< 20% MVC) n = 389** | | **II**  **(moderate,**  **20-50% MVC) n = 340** | | **III**  **(high,**  **> 50% MVC) n = 512** | | **p-value for group effect** | **Effect size*** |
| --- | --- | --- | --- | --- | --- | --- | --- | --- |
|  |  |  |  |  |  | |  |  |
| **VO_2max_ (mL/kg/min)** | **Mean** | **P95** | **Mean** | **P95** | **Mean** | **P95** |  |  |
|  | 47.5(8.4) ^b^ | **62.6** | 49.7 (6.8) ^a,c^ | **62.4** | 48.1 (7.7) ^b^ | **61.5** | <0.001 | 0.207 |
| **Cardiac geometry** |  |  |  |  |  |  | 0.543 | 0.004 |
| Normal (%) | 92.3 |  | 90.6 |  | 90.0 |  |  |  |
| Eccentric hypertrophy (%) | 7.5 |  | 8.8 |  | 8.6 |  |  |  |
| Concentric remodeling (%) | 0 |  | 0.3 |  | 1 |  |  |  |
| Concentric hypertophy (%) | 0.3 |  | 0.3 |  | 0.4 |  |  |  |
|  |  |  |  |  |  |  |  |  |
| **Cardiac dimensions** | **Mean** | **P95** | **Mean** | **P95** | **Mean** | **P95** |  |  |
| LVEF (%) | 61 (7) | **72** | 61 (7) | **72** | 61 (6) | **72** | 0.915 | 0.001 |
| SWT (mm) | 8 (1) ^b^ | **9** | 8 (1) ^a,c^ | **10** | 8 (1) ^b^ | **9** | <0.001 | 0.069 |
| SWT / BSA (mm/m^2^) | 4.6 (0.6) | **5.6** | 4.5 (0.6) ^c^ | **5.5** | 4.6 (0.6) ^b^ | **5.7** | 0.007 | 0.047 |
| LVEDD (mm) | 49 (4) ^b^ | **55** | 50 (4) ^a,c^ | **57** | 49 (4) ^b^ | **56** | <0.001 | 0.069 |
| LVEDD /BSA (mm/m^2^) | 30 (3) | **34** | 29 (2) | **33** | 30 (3) | **35** | <0.001 | 0.029 |
| LVPW (mm) | 7 (1) ^b^ | **9** | 8 (1) ^a,c^ | **10** | 8 (1) ^b^ | **9** | <0.001 | 0.065 |
| LVPW/BSA (mm/m^2^) | 4.5 (0.6) | **5.6** | 4.4 (0.5) ^c^ | **5.3** | 4.6 (0.6) ^b^ | **5.7** | 0.001 | 0.041 |
| LVEDV (mL) | 113 (21) ^b^ | **145** | 121 (22) ^a,c^ | **158** | 113 (21) ^b^ | **152** | <0.001 | 0.066 |
| LVEDV/BSA (mL/m^2^) | 68 (12) | **89** | 70 (10) | **86** | 69 (11) | **89** | 0.206 | 0.066 |
| LV mass (g) | 121 (26) ^b^ | **165** | 134 (31) ^a,c^ | **192** | 124 (30) | **179** ^b^ | <0.001 | 0.106 |
| LV mass / BSA (g/m^2^) | 73 (14) ^b^ | **100** | 77 (15) ^a^ | **103** | 75 (15) | **103** | 0.002 | 0.131 |

Data of LV measures are mean (SD) and 95th (P95) percentile. Abbreviations: BSA, body surface area; SWT, septal wall thickness; LVEDD, left ventricular end diastolic diameter; LVEDV, left ventricular end diastolic volume; LVEF, left ventricular ejection fraction; LVPW, LV posterior wall. Symbols: ^a^ p<0.05 vs. A; ^b^ p<0.05 vs. B; ^c^ p<0.05 vs. C; * assessed with partial eta squared.
