# Supplementary material for: Normative Values for Sport-Specific Left Ventricular Dimensions and Exercise-Induced Cardiac Remodeling in Elite Spanish Male and Female Athletes
Source: Sports Med Open. 2022 Sep 15;8:116. doi: 10.1186/s40798-022-00510-2 (PMC9478009; doi:10.1186/s40798-022-00510-2)
Supplement: Supplementary file 6 — Additional file 6: Pearson’s correlations between maximum oxygen uptake and left ventricular (LV) measures by sex (all sports combined). [file 40798_2022_510_MOESM6_ESM.docx]

**Supplementary file 6**. Pearson’s correlations between maximum oxygen uptake and left ventricular (LV) measures by sex (all sports combined).

|  | Men  (n =2,041) | | Women  (n =1,241) | |
| --- | --- | --- | --- | --- |
| LV measures | r-coefficient | p-value | r-coefficient | p-value |
| LVEF (%) | 0.037 | 0.117 | 0.020 | 0.511 |
| SWT (mm) | 0.095 | <0.001 | 0.173 | <0.001 |
| SWT / BSA (mm/m^2^) | 0.416 | <0.001 | 0.410 | <0.001 |
| LVEDD (mm) | 0.185 | <0.001 | 0.174 | <0.001 |
| LVEDD / BSA (mm/m^2^) | 0.598 | <0.001 | 0.498 | <0.001 |
| LVPW (mm) | 0.089 | <0.001 | 0.162 | <0.001 |
| LVPW / BSA (mm/m^2^) | 0.421 | <0.001 | 0.401 | <0.001 |
| LVEDV (mL) | 0.176 | <0.001 | 0.169 | <0.001 |
| LVEDV / BSA (mL/m^2^) | 0.464 | <0.001 | 0.397 | <0.001 |
| LV mass (g) | 0.166 | <0.001 | 0.213 | <0.001 |
| LV mass / BSA (g/m^2^) | 0.427 | <0.001 | 0.421 | <0.001 |

Abbreviations: BSA, body surface area; SWT, septal wall thickness; LVEDD, left ventricular end diastolic diameter; LVEDV, left ventricular end diastolic volume; LVEF, left ventricular ejection fraction; LVPW, LV posterior wall.
